# Potential Anti-Inflammatory Effects of the Hydrophilic Fraction of Pomegranate (*Punica granatum* L.) Seed Oil on Breast Cancer Cell Lines

**DOI:** 10.3390/molecules19068644

**Published:** 2014-06-24

**Authors:** Susan Costantini, Fabiola Rusolo, Valentina De Vito, Stefania Moccia, Gianluca Picariello, Francesca Capone, Eliana Guerriero, Giuseppe Castello, Maria Grazia Volpe

**Affiliations:** 1 Istituto Nazionale per lo studio e la cura dei tumori “Fondazione Giovanni Pascale”-IRCCS–80131 Napoli, Italy; 2 Istituto di Scienze dell’ Alimentazione-CNR, Via Roma 64, 83100 Avellino, Italy

**Keywords:** anti-inflammatory effects, bioactive molecules, cell viability, cytokines, pomegranate (*Punica granatum* L.)

## Abstract

In this work, we characterized conjugated linolenic acids (e.g., punicic acid) as the major components of the hydrophilic fraction (80% aqueous methanol extract) from pomegranate (*Punica granatum* L.) seed oil (PSO) and evaluated their anti-inflammatory potential on some human colon (HT29 and HCT116), liver (HepG2 and Huh7), breast (MCF-7 and MDA-MB-231) and prostate (DU145) cancer lines. Our results demonstrated that punicic acid and its congeners induce a significant decrease of cell viability for two breast cell lines with a related increase of the cell cycle G_0_/G_1_ phase respect to untreated cells. Moreover, the evaluation of a great panel of cytokines expressed by MCF-7 and MDA-MB-231 cells showed that the levels of VEGF and nine pro-inflammatory cytokines (IL-2, IL-6, IL-12, IL-17, IP-10, MIP-1α, MIP-1β, MCP-1 and TNF-α) decreased in a dose dependent way with increasing amounts of the hydrophilic extracts of PSO, supporting the evidence of an anti-inflammatory effect. Taken together, the data herein suggest a potential synergistic cytotoxic, anti-inflammatory and anti-oxidant role of the polar compounds from PSO.

## 1. Introduction

The scientific world today recognizes the richness of flavonoids, vitamins (A, B and C), tannins and immune-boosting antioxidants in pomegranate (*Punica Granatum* L.), which is a fruit native to tropical and subtropical regions, originated from the Middle East and India, and has been empirically used for centuries for its medicinal purposes [1]. The pomegranate fruit is delimited by a leathery pericarp, embedding numerous *arils*, each a single seed surrounded by a translucent juice-containing sac. Thus, the fruit itself gives rise to three parts: the seeds, about 3% of the weight of the fruit, and themselves containing about 20% oil, the juice, about 30% of the fruit weight, and the peels (pericarp) which also include the interior network of membranes [2]. There is growing interest in this fruit because it is considered to be a functional food of great benefit to the human diet as it contains several groups of substances that are useful for disease risk reduction. For instance, pomegranate is rich in ellagic acid, a substance that appears to be toxic to cancer cells [3]. Moreover, some studies showed a propensity of the pomegranate in mitigating some typical symptoms of menopause such as depression and bone fragility [4]. In general, the potent antioxidant activity of pomegranate is attributed to its polyphenols. Pomegranate polyphenols include flavonoids (flavonols, flavanols, anthocyanins *etc*.) as well as condensed tannins (proanthocyanidins), and hydrolyzable tannins (ellagitannins and gallotannins), all of which are substances able to inactivate the products of the oxidative catabolism that trigger cell disorders, aging as well as numerous cardiovascular diseases. Other phytochemicals identified from the pomegranate are organic and phenolic acids, sterols and triterpenoids, fatty acids, triglycerides, and alkaloids [5].

The antioxidant activity of phenolics is mainly due to their redox properties, which allow them to act as reducing agents, hydrogen donors and metal chelators. In fact, over the past few years, many researchers have found that products derived from pomegranate can be used for the prevention and treatment of certain types of cancers such as colon [4] and prostate cancer [6,7,8]. Similarly, the pomegranate-induced inhibition of the proliferation of breast cancer cells has been documented [9]. The polyphenols contained in the juice, especially anthocyanins and tannins are also capable to quench the effects of ultraviolet (UV) rays, contributing to minimize the primary risk factor of skin cancer [10].

As regards pomegranate seeds, they are a residue obtained from pomegranate juice and contain vitamin E, ellagic acid, sterols and fatty acids, especially high amounts of conjugated linolenic acids such as punicic acid [11]. Pomegranate seed oil (PSO) is often considered a waste product, although it finds a wide range of use as a food ingredient or for lubricants, fuels and additives for paint formulations. In recent years seed oils in general are receiving a growing interest due to their high concentration of both hydrophilic and lipophilic bioactive components, which have a high potential for nutritional, pharmaceutical, and cosmetic purposes [12].

In particular, it has been reported that the hydrophilic components of PSO have a broad spectrum of biological activities, such as antioxidant properties and binding affinity against eicosanoid receptors (e.g., peroxisome proliferator-activated receptors α and γ), by this way regulating gene expression and suppressing chemically induced carcinogenesis [13]. Indeed, these bioactive compounds explicate anticarcinogenic effects by inhibition of cyclooxygenase-2 (COX-2) and lysyl oxidase (LOX) and inhibition of an activation factor, Nuclear Factor-Kappa B (NF-Κβ), thereby decreasing invasion, angiogenesis and metastasis; moreover, these components promote a significant decrease of total hepatic cytochrome P450 content which activates procarcinogens and inhibits tumor initiation; furthermore, pomegranate-derived bioactive compounds inhibit enzymes like carbonic anhydrase and ornithine decarboxylase that are active in cancer cells growth [14]. Relying on mechanisms different from those proposed for pomegranate juice polyphenols, PSO has been proven to exert *in vivo* chemopreventive effects again skin cancer [15].

PSO accounts for 12%–20% of total seed weight, and it mostly consists of triacylglycerols containing approximately 80% of conjugated octadecatrienoic (C_18:3_) fatty acids, with high content of the *cis*-9,*trans*-11,*cis*-13 isomer (*i.e*., punicic acid). Minor components of the oil include sterols, steroids, and cerebrosides [16]. Terpenoids, tocopherols and sitosterols have been also identified in the unsaponifiable fraction of PSO [17].

The purpose of the present investigation was to extract and preliminarily characterize the hydrophilic substances from seed oil of pomegranates grown in the Mediterranean region of Italy and to evaluate their potential effects on several colon (HT29 and HCT116), liver (HepG2 and Huh7), breast (MCF-7 and MDA-MB-231) and prostate (DU145) cancer lines.

## 2. Results and Discussion

### 2.1. Extraction of Hydrophilic Compounds from Pomegranate Seed Oil

Several studies have been performed to evaluate the efficacy of pomegranate products obtained from arils, peel and oils as anti-proliferative, anti-invasive, and pro-apoptotic agents against various cancer cell lines. Adams *et al.* [18] revealed that pomegranate juice suppresses cancer activity through the combined antioxidant and antinflammatory effects by modulating the inflammatory cell signaling in colon cancer cells. Malik *et al.* [19] suggested that pomegranate juice may have cancer chemopreventive as well as cancer-chemotherapeutic effects against prostate cancer in humans. Pomegranate fruit extracts, also including PSO, possess proven antitumor-promoting effects in mouse skin. 

On the contrary, few studies are available about the characterization and evaluation of biological activities of the hydrophilic fraction extracted from PSO. Nevertheless, it can be expected on the basis of the investigations on polyphenols-rich hydrophilic components of plant oils, that the hydrophilic extracts of PSO may exert several health beneficial effects. In this experiments oil was extracted with the Soxhlet method from pomegranate seed, obtaining a yield of 18% of the dry seed mass (DM), which is very similar to that reported previously for Georgia-grown pomegranate seeds [20]. The extraction yield of the hydrophilic fraction, performed with 80% methanol, was about 9% (g extract/100 oil) as shown in Table 1, along with the total content of polyphenols and the antioxidant activity. The total polyphenol content was particularly high (about 23 mg/g oil expressed as gallic acid equivalent) if compared to previous determinations while the *in vitro* antioxidant activity expressed as percentage of inhibition (% I) of DPPH was similar to that previously reported by Jing *et al.* [21].

**Table 1:** Yield, total polyphenol content (TPC) and *in vitro* anti-oxidant properties of the hydrophilic fraction of PSO. TPC is expressed as mg gallic acid equivalent/100g oil; antioxidant activity is expressed as percentage of inhibition (% I) of DPPH. Results are the mean value ± standard deviation.

**Polar Extracts From PSO**	**Yield (%)**	**TPCmg GAE/100 g oil**	**DPPH (I%)**
	8.93 ± 1.02	23.07 ± 1.44	96.80 ± 8.93

**Figure 1:**
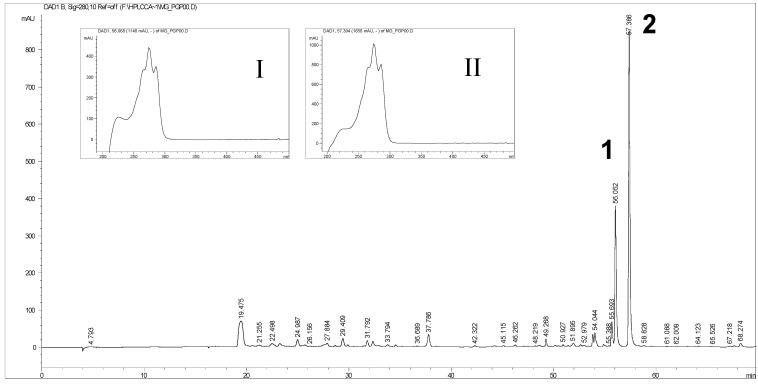
We show RP-HPLC-DAD separation of the polar extracts (80% methanol) from pomegranate oil. The chromatogram was performed at λ = 280 nm. Moreover we report in the insets I and II the UV-Vis spectra evaluated for thepeaks no. 1 and 2 corresponding at two specific retention times, t_R_ = 56.1 min (no. 1) and t_R_ = 57.4 min (no. 2).

The RP-HPLC separation of the polar extracts from pomegranate oil (Figure 1), monitored by UV at λ = 280 nm, exhibited two intense peaks at specific retention times (t_R_) = 56.1 min (no. 1), that was also flanked by a shoulder at t_R_ = 55.7 min, and 57.4 min (no. 2), in addition to a series of minor components at low and intermediate t_R_. Only trace amounts of possible phenol acids, hydroxycinnamic acids and flavonoids occurred into the extracts, as demonstrated by simultaneously monitoring the HPLC separation at λ=320 and 360 nm (not shown). The absence of the most common phenol acids in detectable amounts was confirmed by silica-gel thin layer chromatography (TLC) comparative analysis, using synthetic standards as reference the compounds (*i.e.*, ferulic, vanillic, chlorogenic, gallic and syringic acids).

In the insets of Figure 1 are shown the UV-Vis spectra of the most intense peaks (no. 1 and 2), recorded by the diode array detector (DAD). 

The UV-Vis spectra of the most abundant components exhibited a three-maxima pattern, with absolute λ_max_ centered at 272–274 nm, which strictly matched that expected spectrum of punicic acid (*c*-9, *t*-11, *c*-13-18:3), a conjugated linolenic acid. The less abundant HPLC peak at t_R_ = 55.7 min exhibited a closely similar UV-Vis spectrum (not shown). Hence, no appreciable qualitative differences were observed among the UV-Vis spectra of the three considered HPLC peaks, suggesting that they should be structurally related components, most likely geometrical isomers of punicic acid (e.g., α-eleostearic acid, *c-*9, *t-*11, *t-*13-18:3). More into details, relying on literature data [22], peak abundances, elution order and the measured slight discrepancy among the λ_max_ values, peak no. 1 was assigned to α-eleostearic acid (λ_max_ = 272 nm) and peak no. 2 to punicic acid (λ_max_ = 274 nm). The assignment of the components as free fatty acids was confirmed by separate HPLC analysis of standard C_18_ polyunsaturated fatty acids (e.g., linoleic and conjugated linoleic acids) which eluted at t_R_ very close to peak no. 2. For instance, conjugated linoleic acid (CLA, c9, t11-18:2) eluted at t_R_ = 57.1 min. In this latter case the HPLC runs were monitored also at λ = 220 and 235 nm. 

**Figure 2:**
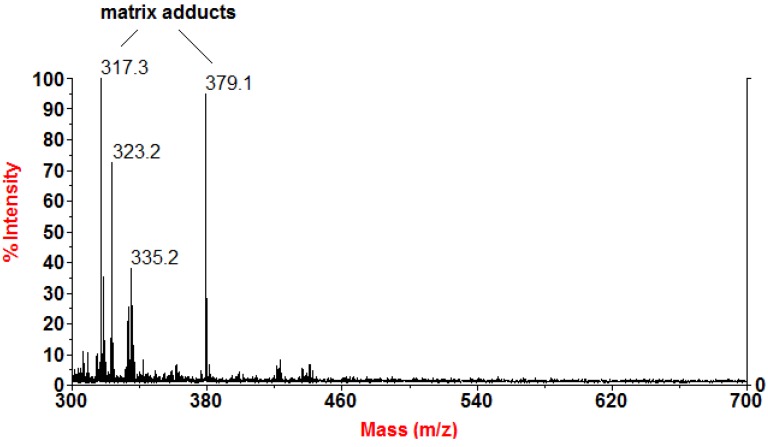
MALDI-TOF MS spectrum of the unfractionated polar extract from pomegranate oil. The signal at *m/z* 323.2 arose from sodium adducts of C_18:3 _carboxylate sodium salts.

To rule out the possible presence of possible diacylglycerols in the polar phase, a MALDI-TOF MS analysis of the unfractionated extract was performed. Although the low molecular mass region generally has a limited informative potential in MALDI-TOF MS due to the matrix interference, a signal at *m/z* 323, missing in the spectrum of the matrix alone (blank acquisition of α-cyano-4-hydroxycinnamic acid), was clearly detected (Figure 2). In MALDI-TOF MS analysis free fatty acids are detected as ion adducts of the carboxylate sodium salts (RCOO-Na + Na^+^). The *m/z* 323 signal corresponded to the sodium adduct of a C_18:3 _fatty acid-sodium salt, thereby confirming the identification of punicic acids and α-eleostearic acid as the main components of the polar extracts from pomegranate oil. No signals were detected for the corresponding mono- and di-acylglycerols (expected *m/z* 375 and 635, respectively, as Na^+^-adducts).

Therefore, the biological activities determined in the current studies, as described below, are for the most to be ascribed to the action of punicic acid and its trienoic fatty acid congeners (conjugated linolenic acids), rather than to the phenolics. Consistently, some recent studies have already assessed the anti-inflammatory activity of dietary punicic acid, that suppresses obesity-related inflammation [23] and prevents or contrasts the inflammatory bowel disease [24]. 

### 2.2. Colorimetric Assay with Sulforhodamine B

**Figure 3:**
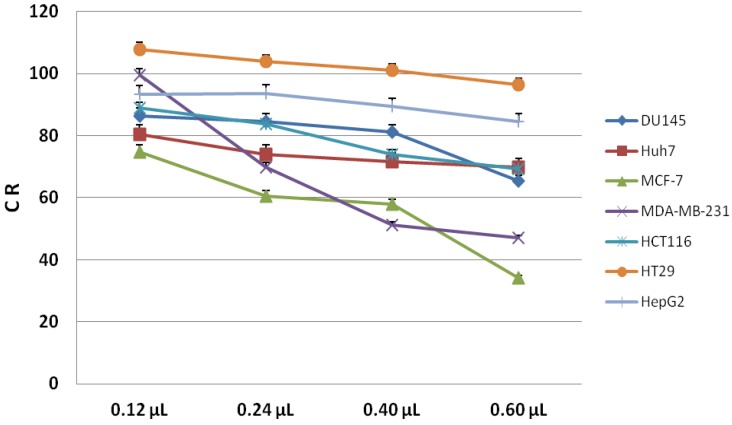
Cell lines growth curves (CR) after 24 h of treatment with hydrophylic extracts of PSO. In details, the viability percentages are reported as mean and standard deviation of triplicate data.

The cell viability of several cancer lines of colon (HT29 and HCT116), liver (HepG2 and Huh7), breast (MCF-7 and MDA-MB-231) and prostate (DU145) was determined after 24 h stimulation with the hydrophilic fraction of PSO in order to identify the IC_50_ concentration, corresponding to the extract amount that causes 50% inhibition of cell growth (Figure 3). The cell viability of untreated cells was used as control.

After 24 h of challenge, HT29, HepG2, HuH7, HCT116 and DU145 cells retained a relatively constant viability at increasing dose of hydrophilic extract from PSO as evidenced by the overlapping of growth curves in Figure 3. The decrease in cell viability (evaluated as means and standard errors) became significant for two breast cell lines, MDA-MB-231 and MCF-7, which reached IC_50_ at doses of 0.60 μL and 0.50 μL extracts, respectively. 

### 2.3. Apoptosis Assay

Subsequently, we evaluated the ability of hydrophilic extracts from PSO to induce apoptosis in two breast cell lines, namely MDA-MB-231 and MCF-7, whose viability was mostly affected by stimulation. In particular, it has been calculated the rate of apoptosis by classifying four cell populations:

1. Live cells which are not undergoing to apoptosis: annexin V (−) and cells dead marker (−);
2. Cells in early apoptosis: annexin V (+) and cells dead marker (−);
3. Cells in adavanced apoptosis: annexin V (+) and cells dead marker (+);
4. Dead cells which do not cross the apoptotic process (necrosis): annexin V (−) and cells dead marker (+).

In details, 7-aminoactinomycin D (7-AAD) was used as the marker of cell death. The treatment with different amounts of extracts did not increase significantly the apoptosis in both breast cell lines (Table 2A). This was evidenced by the increase of cell density in the plot of live cells and by the presence of few cells in early apoptosis/death and, even further, in advanced apoptosis status.

Table 2Rate of apoptosis (**A**) and cell percentages in G_0_/G_1_, S and G_2_/M phases (**B**) evaluated in two breast cell lines, MDA-MB-231 and MCF-7, non treated and treated for 24 h with 0.5 μL and 0.6 μL of hydrophilic extracts from PSO, respectively. Mean of triplicate assays are reported.

**Table:** A

Cells	Live Cells (%)	Cells in Early Apoptosis (%)	Cells in Late Apoptosis (%)	Dead Cells (%)
**MCF-7 non treated**	95.30 ± 1.02	0.95 ± 0.02	3.35% ± 1.21%	0.40 ± 0.04
**MCF-7 treated **	92.85 ± 1.15	3.50 ± 0.77	2.65% ± 0.07%	1.00 ± 0.09
**MDA-MB-231 non treated**	96.79 ± 1.09	1.31 ± 0.42	1.86% ± 0.54%	0.05 ± 0.02
**MDA-MB-231 treated **	95.75 ± 2.04	2.95 ± 0.61	1.10% ± 0.05%	0.20 ± 0.09

**Table:** B

Cells	G_0_/G_1 _ (%)	S (%)	G_2_/M (%)
**MCF-7 Non treated**	5.0 ± 1.2	94.2 ± 6.4	0.8 ± 0.1
**MCF-7 treated **	79.2 ± 5.3	7.9 ± 1.6	12.9 ± 2.1
**MDA-MB-231 non treated**	20.8 ± 3.6	18.9 ± 1.2	60.3 ± 4.6
**MDA-MB-231 treated **	67.5 ± 6.1	19.7 ± 2.1	12.8 ± 2.3

### 2.4. Cell Cycle Assay

We evaluated the cell cycle in MCF-7 and MDA-MB-231 cell lines treated with the extract. In particular, increasing doses of the extract slowed down the cell cycle G_0_/G_1_ phase in MCF-7 if compared to untreated cells (Table 2B), with a slight lengthening of G_0_/G_1_ phase peak and a shortening of S and G_2_/M phase peaks. In fact, the cell percentages in G_0_/G_1_, S and G_2_/M phases were 5.0%, 94.2% and 0.8% in untreated cells and 79.2%, 7.9% and 12.9% in cells treated with 0.5 μL of extracts. Moreover, a very similar trend was obtained for MDA-MB-231 that underwent a slowdown of the cell cycle G_0_/G_1_ phase compared to untreated cells with the following percentages in G_0_/G_1_, S and G_2_/M phases: 20.8%, 18.9% and 60.3% in untreated cells and 67.5%, 19.7% and 12.8% in cells treated with 0.6 μL of extracts.

### 2.5. Evaluation of Cytokine Levels on MDA-MB-231 and MCF-7 Supernatants

**Figure 4:**
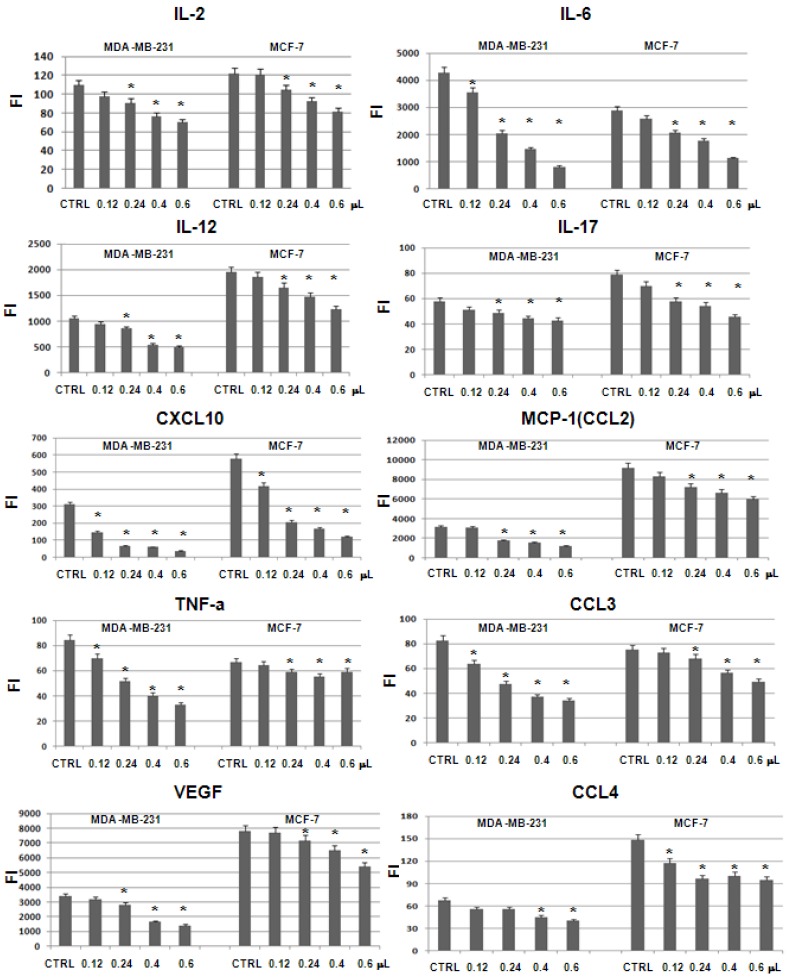
Cytokine levels (expressed as fluorescence intensity) evaluated in MCF-7 and MDA-MB-231 cells after treatment with hydrophilic extracts of PSO performed in triplicate. We indicate with * the comparisons between the levels in the untreated and treated cells that resulted statistically significant (*p* < 0.05 by T-test).

The cytokine florescence intensities were evaluated in the MDA-MB-231 and MCF-7 supernatants after 24 h incubation with the hydrophilic extracts from PSO. The results obtained were compared with untreated cells used as control. These experiments showed similar results in the two breast cancer cell lines. In particular, the levels of vascular endothelial growth factor (VEGF) and nine pro-inflammatory cytokines, such as IL-2, IL-6, IL-12, IL-17, CXCL10, MIP-1α (CCL3), MIP-1β (CCL4), MCP-1 (CCL2) and TNF-α, decreased in dose dependent fashion with increasing levels of the extract (Figure 4). Levels of all these ten cytokines have been reported as significantly increased in breast cancer patients as compared to controls and indicated as markers of tumor and metastatic progression [25].

MCP-1 is a direct mediator of angiogenesis, inducing *in vitro* endothelial cell migration, endothelial cell sprouting from aortal rings in the absence of an inﬂammatory response, and *in vivo* angiogenesis in the matrigel plug assay. It has been reported that MCP-1 expression is activated from NF-kB and the inhibition of its activity resulted in a signiﬁcant prolongation of the survival of immunodeﬁcient mice bearing human breast carcinomas [26]. In addition, IL-17 is a pro-inflammatory interleukin that stimulates fibroblasts as well as epithelial and endothelial cells, macrophages and keratinocytes to produce some chemokines including MCP-1 and some cytokines such as IL-6 and TNF-α. The function of IL-17 is essential to a subset of CD4+T cells called T helper 17 (Th17) whose role is connected to many immune and autoimmune diseases [27]. 

TNF-α is a pro-inflammatory cytokine that plays an important role in apoptosis, proliferation, cell differentiation and viral replication. At increased levels, TNF-α induce the activation of anti-apoptotic transcription factor NF-kB, which once moved from the cytoplasm to the nucleus, promotes cell survival and tumor progression [28]. On the other hand, IL-6 is one of the most important mediators of the acute phase response and acts as both a pro-inflammatory and anti-inflammatory cytokine. This interleukin regulates TNF-α activity and stimulates the secretion of VEGF in cancer cells being an established potent angiogenesis factor with pro-inflammatory properties [29].

Therefore, since MCP-1 expression is activated from NF-kB and TNF-α induces NF-kB activation, we can hypothesize that their reduction can inhibit the activation of NF-kB by blocking tumor growth. However, further studies will regard the evaluation of NF-kB after 24 h treatment of MDA-MB-231 and MCF-7 cells with hydrophilic extracts of PSO to verify our hypothesis.

MIP-1α (CCL3) and MIP-1β (CCL4) are two chemokines crucial for immune responses towards infection and inflammation. They activate human granulocytes (neutrophils, eosinophils and basophils) which can lead to acute neutrophilic inflammation, and also induce the synthesis and release of other pro-inflammatory cytokines such as IL-6 and TNF-α from fibroblasts and macrophages [30]. These data evidence that the decreased levels of MIP-1α and MIP-1β as well as those of IL-6, IL-17, MCP-1, TNF-α and VEGF are certainly mutually correlated.

IL-12 is an interleukin, naturally produced by dendritic cells, macrophages and human B-lymphoblastoid cells in response to antigenic stimulation. It stimulates, from T and natural killer (NK) cells, the production of TNF-α and interferon-gamma (IFN-γ), which in turn increases the production of CXCL10 that mediates this anti-angiogenic effect. Therefore, this aspect can explain why both IL-12 and IP-10 decreased after challenging MDA-MB-231 and MCF-7 cells with hydrophilic extract of PSO. 

Finally, IL-2 is a pro-inflammatory cytokine, necessary for the growth, proliferation, and differentiation of T cells and is used in many clinical trials as an immunotherapy for the treatment of cancers [31]. Hence its down-regulation (or under-expression) can arrest cancer progression.

### 2.6. Interactomic Studies

**Figure 5:**
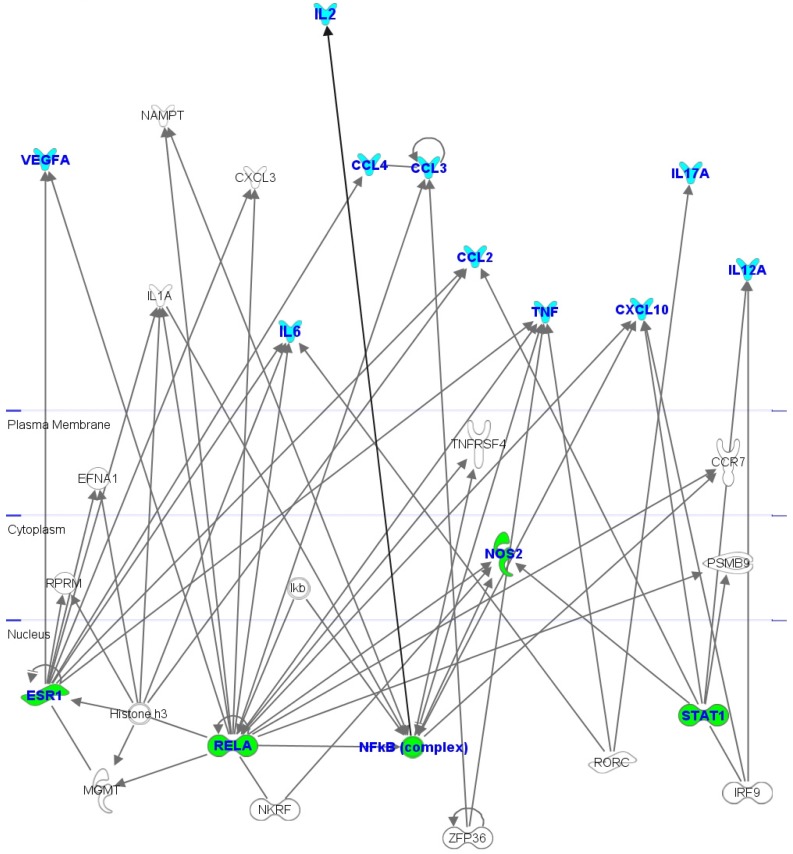
Interactomic analysis by Ingenuity Pathway Analysis (IPA) of significant molecules. The interactome shows the close functional association between significant cytokines (evidenced with cyan symbols) as well as the paths in which other functionally relevant molecules are also involved (evidenced with white symbols). In particular, some hub node (ESR1, NF-kB, NOS2, RELA subunit, and STAT1) are evidenced by green symbols.

All of the ten analyzed significant cytokines are involved in a network named “Inflammatory response, Cell to cell signaling and interaction, Hematological System Development and Function” on the basis of associated functions and data mining from experimental studies reported in literature (Figure 5). This network highlights that these proteins are connected with some hub nodes such as NF-kB subunits (NFκB complex and RELA), STAT1 (signal transducer and activator of transcription 1), RORC (RAR related orphan receptor C), and ESR1 (estrogen receptor 1) that is one of clinical breast cancer markers commonly used [32]. In details, one can see that (i) IL-2, IL-6, CXCL10, MIP-1α (CCL3), TNF-α and VEGF are connected with NF-κB subunits (NFκB complex and RELA); (ii) IL-12, CXCL10 and MCP-1 (CCL2) with STAT1; (iii) IL-6, IL-17 and TNF-α with RORC; and (iv) CCL2, CCL4, TNF-α and VEGF with ESR1.

It is important to underline that NF-κB subunits and STAT1 are connected with NOS2 (nitric oxide synthase 2). In literature it is reported that both STAT1 activation and NF-κB induce the production of NOS2 during an inflammatory response and the related production of nitric oxide (NO), a highly reactive free radical with important second messenger functions involving the mediation of inflammatory events. Hence the increased expression of NOS2 and NO levels was evidenced in several inflammatory diseases and, in particular, was demonstrated to affect survival of breast cancer patients [33].

Therefore our interactomic studies evidence that these ten significant cytokines can be mutually interconnected, and the decrease of their levels can induce NF-κB and STAT1 inactivation in addition to the modulation of NOS2 and the related NO production. Hence, overall these data suggest a potential synergic anti-inflammatory and anti-oxidant role of the hydrophilic compounds from PSO. 

## 3. Experimental

### 3.1. Samples and Chemicals

The pomegranate (*Punica granatum* L.) seeds were obtained from mature fruits grown in the Campania region (Southern Italy). Ripe fresh fruits were harvested during October and November from different trees randomly selected in order to be representative of the whole plantation. Fruits were transported to the laboratory and approximately n = 30 of pomegranates at harvesting maturity was selected. The seeds were manually separated from the pulp and subsequently lyophilized and ground in a domestic grinder. The obtained seed flour was stored at −20 °C until analysis. All chemicals and solvents used were purchased from Carlo Erba (Milano, Italy) and Sigma-Aldrich Co LLC (Munich, Germany) and were of analytical grade. 

### 3.2. Oil Extraction

The oils were extracted with the Soxhlet method according to the Methods of Analysis Recommended for Chemical Control of Foods by the National Institute of Health, 1996 (Reports ISTISAN 96/34). A sample of dried ground seeds (20 g) was extracted using diethyl ether (250 mL) as solvent, for 14 h at 270 °C. The oil was recovered after distilling the solvent off with a rotary evaporator (Mod. Hei VAP Value, Heidolph, Bergamo, Italy), dried to constant weight in a vacuum chamber and finally weighed. 

### 3.3. Extraction and Determination of Total Phenolics

Oil sample (10 g) was diluted in 80% aqueous methanol (10 mL), magnetically stirred for 30 min, at room temperature (25 °C) in the dark and finally filtered. After separation of the phases, the lower layer was collected; the process was repeated three times, the polar phases were pooled and evaporated. The total phenolics content was measured spectrophotometrically at 765 nm using the Folin–Ciocalteu reagent (FCr) method [34]. The extracts were dissolved in ethanol and then diluted with distilled water to a concentration of 0.075 mg/mL of the hydrophilic extracts; the ethanol volume did not exceed 10 % of the final solution. Next, the sample solutions (2 mL) were placed into test tubes and mixed with a 1:10 aqueous dilution of FCr (10 mL) and then an anhydrous sodium carbonate solution (8 mL, 75 mg/mL). All reactions were kept in semidarkness for 5 min in a water bath (40 °C). The absorbance was then recorded (760 nm) with a UV-Vis spectrophotometer (model U-3010, Hitachi, Tokyo, Japan). The total phenolic content was expressed as gallic acid equivalents (GAE) using the gallic acid standard curve (5–50 μg/mL). 

### 3.4. RP-HPLC-DAD Analysis

The reversed phase-high performance liquid chromatography (RP-HPLC) analyses were carried out using an Agilent 1100 HP Series (Agilent Technologies, Palo Alto, CA, USA) modular system equipped with a dyode array detector (DAD). The stationary phase was a 250 × 2.1 mm i.d. C18 reversed-phase column, particle diameter 4 μm (Phenomenex, Torrance, CA, USA). The polar extract of PSO (20 μL) was ten-fold diluted with 0.1% TFA and separated at a constant flow-rate of 0.2 mL/min applying the following gradient of solvent B (acetonitrile/0.1% trifluoroacetic acid, TFA): isocratic elution at 4% B for 5 min, 4%–60% B in 5–45 min, and finally 60%–100% B in 45–60 min. Solvent A was 0.1% TFA in HPLC-grade water. Analyses were carried out in triplicate. 

### 3.5. MALDI-TOF MS Analysis

Matrix assisted laser desorption ionization-time of flight (MALDI-TOF) mass spectrometry (MS) experiments were carried out using a Voyager DE-Pro instrument (Perseptive Biosystem, Framingham, MA, USA) equipped with an N_2_ laser. MS spectra were acquired in the reflector positive ion mode. Matrix was α-cyano-4-hydroxycinnamic acid, prepared by dissolving the crystalline powders (10 mg/mL) in 50% acetonitrile (v/v)/5 mM Na-acetate. Spectra were elaborated with the Data Explorer software 4.0 purchased with the instrument.

### 3.6. Antioxidant Activity (DPPH Assay)

The *in vitro* antioxidant activity of 80% methanol extract of the PSO was evaluated by measuring scavenging properties of the free radical 2,2-diphenyl-1-picrylhydrazyl (DPPH), according to the method of Brand-Williams *et al.* [35]. Polar extract of PSO (500 μL) was mixed with *in situ* generated 0.2 mM DPPH (500 μL) and the absorbance of sample were measured at 517 nm until the values reached the plateau (1h). The antiradical activity was expressed as percentage of inhibition (% I) of the sample (As) compared to the initial concentration of DPPH (Ac) according to the formula: I% = (Ac − As/Ac) × 100.

### 3.7. Cell Culture

Human cancer cell lines, HuH7, HepG2, MCF-7 and HT29 were cultured and expanded at 37 °C in a humidified atmosphere of 5% CO_2_ in culture medium DMEM (Dulbecco’s Modified Eagle’s Medium, Lonza, Verviers, Belgium), supplemented with FBS (Invitrogen, Camarillo, CA, USA) at 10%, penicillin/streptomycin 100× (Euroclone, Devon, UK), Glutamax 100× (Invitrogen) and non-essential amino acids 100× (Invitrogen). Human cancer cell lines DU145, MDA-MB-231 and HCT116 were cultured and expanded in humidified 37 °C/5% CO_2_ incubator in culture medium RPMI1640 w/o l-glutamine (Lonza, Verviers, Belgium), supplemented with FBS (Invitrogen, Camarillo, CA, USA) at 10%, penicillin/streptomycin 100× (Euroclone, Devon, UK), Glutamax 100× (Invitrogen) and non-essential amino acids 100× (Invitrogen). Phosphate buffer (PBS phosphate buffered saline Ca^2+^ and Mg^2+^ free) and trypsin (Ca^2+^ and Mg^2+^ free) were supplied by Euroclone.

### 3.8. Colorimetric Assay with Sulforhodamine B

Cell proliferation was assessed in presence and absence of the methanol extract from PSO by colorimetric assay with sulforhodamine B (SRB, Sigma Aldrich). The cells (3.5 × 10^−4^–4 × 10^−4^) were seeded in 96-multiwell plates in 200 μL of culture medium, and left to grow for 24h at 37 °C for allowing adhesion. Then, the cells were treated with varying amounts of the extract: 0.12 μL (0.4 mg/L), 0.24 μL (0.4 mg/L), 0.4 μL (0.4 mg/L), 0.6 μL (0.4 mg/L), and then incubated for 24 h. The dissolution of the extract was improved by 100 mM dimethyl sulfoxide (DMSO, Sigma Aldrich). From this stock solution, dilutions were made to obtain the different amounts with a final concentration of 0.05% DMSO. Control cultured cells were incubated with the same volume of the solvent. Then, the cells were fixed by the addiction of 10% trichloroacetic acid (Sigma Aldrich) for at least 1 h at 4 °C. Subsequently, the cells were washed with distilled water and air dried. SRB (100 μL) was added to each well and the plate was incubated for 30 min at room temperature in the dark. To remove the dye excess the cells were washed with 1% acetic acid. The number of viable cells was directly proportional to the protein bound-dye formation which was then solubilized with 100 μL of 10 mM Tris base solution pH 10.5, shaking the plates for at least 15 min on a orbital shaker to homogenize the dye solution. Measure of OD was performed by using an automated 96-well plate reader (Microplate Reader, Bio-Rad, Hercules, CA, USA) at λ = 540 nm. All experiments were performed in triplicate and were repeated for three times. The cellular viability was estimated as % compared to untreated cells.

### 3.9. Apoptosis Assay

The cells (1 × 10^6^) have been harvested and twice washed with cold PBS. Subsequently, the cells have been labeled by annexin V & Dead Cell Assay kit, Merck Millipore, Darmstadt, Germany) according to instructions of producer. This assay is based on the detection of phosphatidylserine (PS) on the surface of apoptotic cells using annexin V FITC conjugated in combination with the cell death marker 7-AAD. This staining allows quantitative analysis of live cells, cells in early and later apoptosis and dead cells. The samples have been tested with the Muse ™ Analyzer (Merck Millipore) and were analyzed by a software supplied by the same company.

### 3.10. Cell Cycle Assay

The Muse™ Cell Cycle Assay uses a premixed reagent which includes the nuclear DNA intercalating stain propidium iodide (PI) and RNAse A in a proprietary formulation. PI discriminates cells at different stages of the cell cycle, based on differential DNA content in the presence of RNAse to increase the specificity of DNA staining. The samples were centrifuged at 300*×* *g* for 5 min and after removing and discarding the supernatant, an appropriate volume of PBS was added to each tube (1 mL of PBS per 1 × 10^6^ cells). After centrifugation and removing of the supernatant, 1 mL of ice cold 70% ethanol was added to the resuspending cell pellet in the residual PBS. The tubes were capped and freezed at −20 °C for at least 3 h prior to staining. Ethanol-fixed cells were centrifuged at 300 ×*g* for 5 min at room temperature and the pellet was resuspended in PBS. The cells were centrifuged again at 300 ×*g* for 5 min at room temperature, the supernatant was removed and discarded and cell pellet was resuspended in 200 μL of Muse™ Cell Cycle Reagent and incubated for 30 min at room temperature, in the dark. Cell suspension samples were transferred to a 1.5-mL microcentrifuge tube prior to analysis. 

### 3.11. Bio-Plex Assay

In our approach, the levels of several cytokines, chemokines and growth factors were evaluated at the same time by the BioPlex assay. The multiplex biometric ELISA-based immunoassay, containing dyed microspheres conjugated with a monoclonal antibody specific for a target protein was used, according to the manufacturer’s instructions (Bio-Plex Bio-Rad), to evaluate the levels of different cytokines by Human Cytokine 27-Plex Panel after 24h of incubation with polyphenols in MDA-MB-231 and MCF-7 supernatants. In particular, the following cytokines were evaluated: fourteen interleukins (IL), IL-1β, IL-1ra, IL-2, IL-4, IL-5, IL-6, IL-7, IL-8, IL-9, IL-10, IL-12 (p70), IL-13, IL-15, IL-17, and eotaxin (CCL11), basic fibroblast growth factor (FGF), granulocyte colony-stimulating factor (G-CSF), granulocyte macrophage colony-stimulating factor (GM-CSF), interferon-gamma (IFN-γ), chemokine (C-X-C motif) ligand 10 (CXCL10), monocyte chemo-attractant protein-1 (MCP-1), two macrophage inflammatory protein–1s, MIP-1α and MIP-1β, platelet-derived growth factor-β (PDGF-β), RANTES (regulated on activation, normal T cell expressed and secreted), tumor necrosis factor-α (TNF-α) and VEGF. Each experiment was performed in duplicate as previously described [17,36]. Protein levels were determined using a Bio-Plex array reader (Luminex, Austin, TX, USA) that quantitates multiplex immunoassays in a 96-well format with very small fluid volumes. The analyte level was calculated using a standard curve, with the Bio-Plex Manager Software provided by the manufacturer.

### 3.12. Bioinformatics Analysis

The expression level of cytokines in the MDA-MB231 and MCF-7 supernatants after 24 h of incubation with hydrophilic extracts were compared by T-test. Values of *p* < 0.05 were considered to be statistically significant. The statistical program Prism 4 (GraphPad Software, San Diego, CA, USA) was used. Moreover, the possible interactions between the significant proteins were analyzed by Ingenuity Pathway Analysis (IPA).

## 4. Conclusions

In this work, we identified punicic acid and its congeners as the most abundant compounds of the 80% aqueous methanol extract from PSO of Southern Italy and tested their effects on some cancer lines of colon (HT29 and HCT116), liver (HepG2 and Huh7), breast (MCF-7 and MDA-MB-231) and prostate (DU145). The results indicated that the extract: (i) significantly decreased cell viability for two breast cell lines, MDA-MB-231 and MCF-7; (ii) did not significantly increase apoptosis of these two breast cell lines; (iii) increased the cell cycle G_0_/G_1_ phase respect to untreated cells; and (iv) at increasing amounts, decreased the levels of VEGF and nine pro-inflammatory cytokines (IL-2, IL-6, IL-12, IL-17, CXCL10, MIP-1α, MIP-1β, MCP-1 and TNF-α). Our interactomic analysis evidenced that the expression of these ten significant cytokines can be inter-correlated, and their down-regulation can induce NF-kB and STAT1 inactivation and also the modulation of NOS2 and the related NO production. Overall, our data suggest a potential synergistic cytotoxic and anti-inflammatory and anti-oxidant effect of the hydrophilic fraction of PSO on breast cancer cell lines.
